# P-541. Impact of Viral Co-Detections on Respiratory Syncytial Virus (RSV) Viral Loads and Clinical Outcomes in Pediatric Patients

**DOI:** 10.1093/ofid/ofaf695.756

**Published:** 2026-01-11

**Authors:** Rosa Maria Singla Mila, Helena Brenes-Chacon, Marie Wehenkel, Octavio Ramilo, Asuncion Mejias

**Affiliations:** St Jude Children's Research Hospital, Barcelona, Catalonia, Spain; St. Jude Children's Research Hospital, Germantown, TN; St. Jude Children's Research Hospital, Germantown, TN; St. Jude Children's Research Hospital, Germantown, TN; St Jude Children's Research Hospital, Barcelona, Catalonia, Spain

## Abstract

**Background:**

RSV is a major cause of lower respiratory tract infections (LRTI) in infants and young children worldwide. While viral co-detections (V-codt) are common in children with RSV LRTI, their impact on clinical outcomes and RSV viral loads is poorly understood.

**Fig 1.**
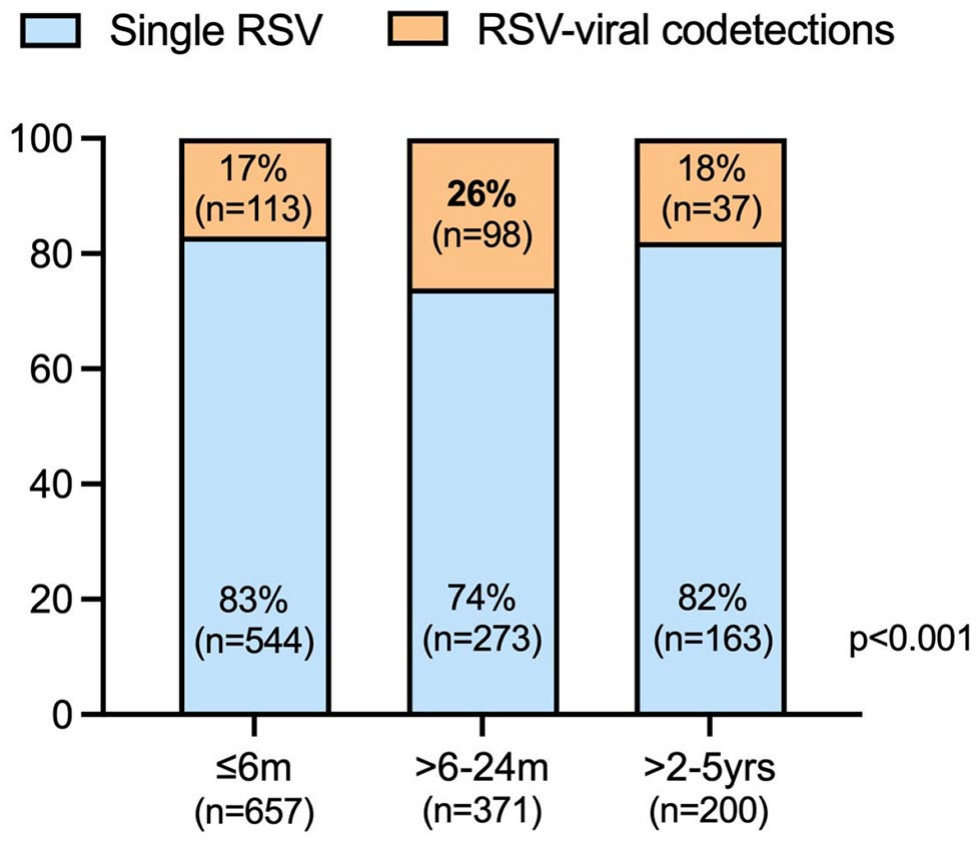
Frequency of single RSV infections vs RSV/viral codetections according to age Proportions of single RSV infections (light blue) and RSV/viral codetecions (orange) are represented in the Y axis. The X axis include the three age categories: ≤6 months, >6–24 months, and >2–5 years. Proportions analyzed by Chi-square (p < 0.001).

**Table 1:**
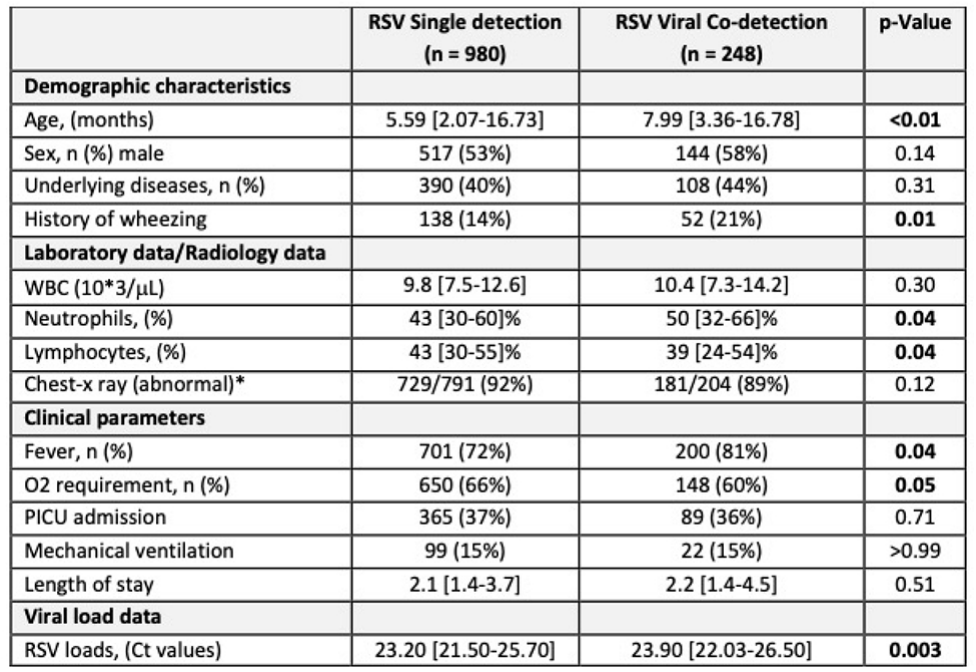
Characteristics of children with single RSV and RSV/viral codetection Abnormal chest X-ray included bronchial wall thickening, atelectasis or consolidation. Continuous variables are expressed as medians 25–75% interquartile ranges (IQR) and categorical data as frequency (%). Numbers in bold indicate significant p values.

**Methods:**

Retrospective cohort of children < 5 years hospitalized with RSV-LRTI at a large pediatric hospital during six respiratory seasons. RSV was diagnosed per standard of care using rt-PCR which provided semiquantitative viral loads (cycle threshold (C_T_) values). Individual PCRs for rhinovirus (RV), adenovirus (ADV), parainfluenza virus (PIV), influenza virus A/B (FluV), and human metapneumovirus (hMPV) were run in parallel as part of a panel. Clinical data was reviewed. Analyses were performed to evaluate the impact of V-codt on RSV loads and on clinical disease severity stratified by age in ≤ 6 months (mo); > 6-24 mo and > 2-5 years (yrs)

**Fig 2.**
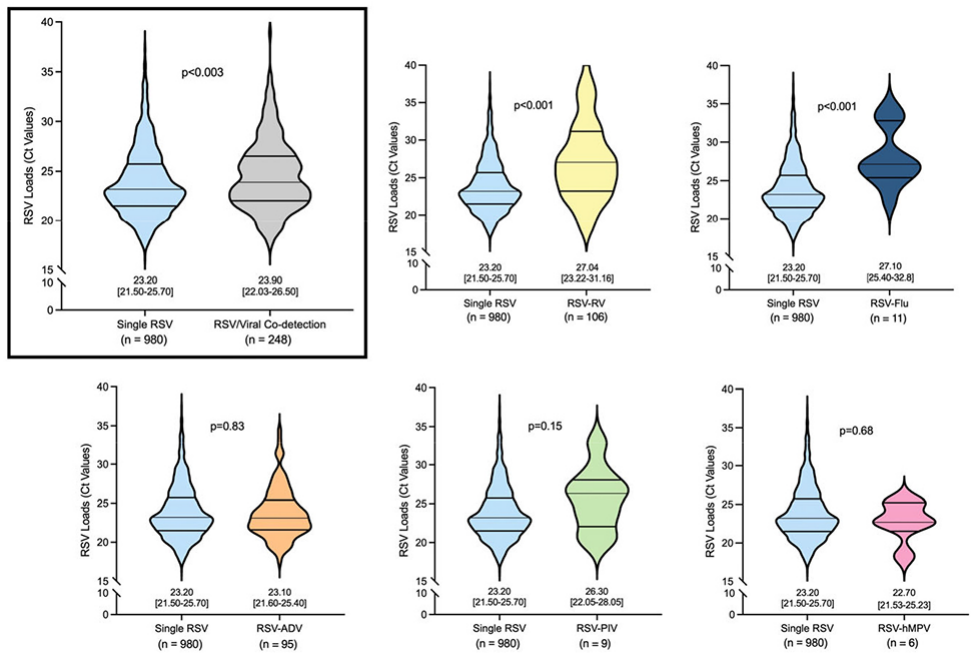
Differences in RSV viral loads between children with single RSV infection vs RSV/Viral Co-Detections. Viral loads Viral loads are expressed as cycle threshold (Ct) values in the Y axis for the six violin lots, that include 25–75% interquartile ranges (IQR) and median values. Viral loads for children with single RSV infection are color coded in blue, for the overall cohort in grey (upper L figure); in yellow for rhinovirus (RV; upper middle figure); dark blue for influenza A/B (Flu; upper R figure); orange for adenovirus (ADV; lower L figure); green for parainfluenza viruses (PIV; lower middle figure); and pink for human metapneumovirus (hmpv; lower R figure). Numbers underneath each plot indicate the median 25%-75% IQR values. Numbers under the X axis labels indicate the number of children in each group. Pairwise comparisons by Mann–Whitney U test (p < 0.05).

**Results:**

Between 2012-18, we identified 1,228 children with RSV LRTI who underwent testing using the viral panel: 54% ≤ 6 mo, 30% >6-24 mo, 16% > 2-5 yrs. RSV alone was identified in 980 children (80%) and RSV/V-codt in 248 (20%). The most common V-codt were RV (n=106; 8.6%) and ADV (n=95; 7.7%) followed by Flu (n=11; 0.9%), PIV (n=9; 0.7%) and hMPV (n= 6; 0.5%). RSV/V-codt were more common in children >6-24 mo (26%) *vs* infants < 6 mo (18%) and children > 2-5yrs (17%; p< 0.001) (Fig 1). Overall, children with RSV/V-codt had fever more frequently (81% vs 72%; p= 0.04) and higher percentage of peripheral blood neutrophils, with no differences in parameters of disease severity (Table 1). In addition, RSV loads were lower in children with RSV/V-codt vs single RSV infection (p< 0.01). Analyses according to specific RSV/viral combinations showed that differences in RSV loads remained significant only for RSV/RV and RSV/FluV (Fig 2).

**Conclusion:**

Concomitant respiratory viruses were identified in one quarter of children with RSV LRTI, were more frequent in older infants, and associated with fever, but not with other clinical parameters. RSV loads were also lower in the context of RSV/viral co-detections. These observations underscore the need to understand RSV-viral interactions, particularly in the context of current clinical studies assessing the effectiveness of preventive and therapeutic interventions for RSV.

**Disclosures:**

Octavio Ramilo, MD, Merck: Advisor/Consultant|Merck: Grant/Research Support|Merck: Honoraria|Moderna: Advisor/Consultant|Pfizer: Advisor/Consultant|Pfizer: Honoraria|Sanofi: Advisor/Consultant Asuncion Mejias, MD, PhD, MsCS, Enanta: Advisor/Consultant|Merck: Grant/Research Support|Moderna: Advisor/Consultant|Pfizer: Advisor/Consultant|Sanofi-Pasteur: Advisor/Consultant

